# Mapping Genetically Controlled Neural Circuits of Social Behavior and Visuo-Motor Integration by a Preliminary Examination of Atypical Deletions with Williams Syndrome

**DOI:** 10.1371/journal.pone.0104088

**Published:** 2014-08-08

**Authors:** Fumiko Hoeft, Li Dai, Brian W. Haas, Kristen Sheau, Masaru Mimura, Debra Mills, Albert Galaburda, Ursula Bellugi, Julie R. Korenberg, Allan L. Reiss

**Affiliations:** 1 Center for Interdisciplinary Brain Sciences Research (CIBSR), Stanford University School of Medicine, Stanford, CA, United States of America; 2 Department of Neuropsychiatry, Keio University, School of Medicine, Tokyo, Japan; 3 Department of Pediatrics & The Center for Integrated Neuroscience and Human Behavior, The Brain Institute, University of Utah, Salt Lake City, UT, United States of America; 4 Department of Psychology, University of Georgia, Athens, GA, United States of America; 5 School of Psychology, Bangor University, Gwynedd, United Kingdom; 6 Department of Neurology, Beth Israel-Deaconess Medical Center, Harvard Medical School, Cambridge, MA, United States of America; 7 Laboratory for Cognitive Neuroscience, Salk Institute for Biological Studies, La Jolla, CA, United States of America; 8 Departments of Radiology and Pediatrics, Stanford University School of Medicine, Stanford, CA, United States of America; Instituto Gulbenkian de Ciência, Portugal

## Abstract

In this study of eight rare atypical deletion cases with Williams-Beuren syndrome (WS; also known as 7q11.23 deletion syndrome) consisting of three different patterns of deletions, compared to typical WS and typically developing (TD) individuals, we show preliminary evidence of dissociable genetic contributions to brain structure and human cognition. Univariate and multivariate pattern classification results of morphometric brain patterns complemented by behavior implicate a possible role for the chromosomal region that includes: 1) GTF2I/GTF2IRD1 in visuo-spatial/motor integration, intraparietal as well as overall gray matter structures, 2) the region spanning ABHD11 through RFC2 including LIMK1, in social cognition, in particular approachability, as well as orbitofrontal, amygdala and fusiform anatomy, and 3) the regions including STX1A, and/or CYLN2 in overall white matter structure. This knowledge contributes to our understanding of the role of genetics on human brain structure, cognition and pathophysiology of altered cognition in WS. The current study builds on ongoing research designed to characterize the impact of multiple genes, gene-gene interactions and changes in gene expression on the human brain.

## Introduction

Imaging genetics research provides an unprecedented opportunity for studying interactions among genes, brain and behavior in humans. For example, studies have explored associations of common genetic polymorphisms, including those related to catechol-*O*-methyl transferase (*COMT*), monoamine oxidase A (*MAOA*) and serotonin transporter length polymorphism (*5-HTTLPR/SLC6A4*), with brain structure and function [1]. Similarly, examination of rare, atypical duplications and deletions associated with disorders such as Williams-Beuren syndrome (WS) can further illuminate our understanding of gene-brain-behavior relationships [2].

WS is a neurodevelopmental disorder caused by a hemizygous deletion of approximately 28 genes on 7q11.23 [3]. WS is associated with poor visuo-spatial construction and increased social drive [4]. The existence of this well delineated profile and known genetic architecture of WS offers unique opportunities to investigate the neurogenetic basis of cognition in humans [5]. Using this approach comparing WS to typically developing (TD) controls, studies have found the genes deleted in WS to be important for intraparietal sulcus (IPS) morphology, which in turn mediates visuo-spatial construction [6], and amygdala-orbitofrontal (OFC)-fusiform circuitry as related to socio-emotional abilities [7]–[9]. These studies suggest genetically controlled neural circuitries for regulating human behavior, and show how brain imaging data may serve as ideal intermediate endophenotypes mediating gene and behavior.

**Figure 1:**
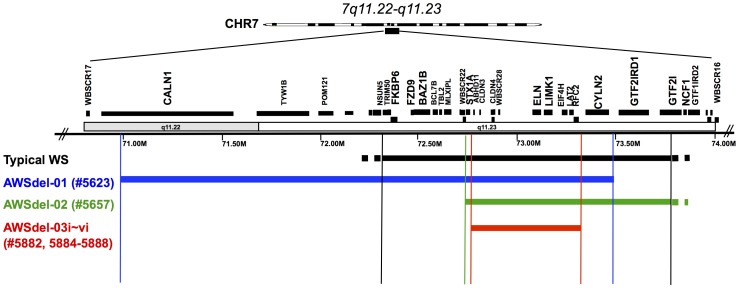
Schematic diagram of deleted genes in WS and in partial deletion participants (AWSdel). Genes listed in the figure are either ones known to be expressed in the brain and are important for neurodevelopment, synaptic plasticity and neuronal reorganization: *LIMK1* [54], *FZD9* [55], *STX1A* [56], *CYLN2* [57], *GTF2 I* [58] and *GTF2IRD1* [59], or are break-points.

To further gain a better understanding of the neurogenetic basis of human behavior using this ‘model disease’ approach, the current study undertook a targeted investigation of persons with WS having rare atypical deletions (AWSdel) by comparing these individuals to WS and TD groups. While most individuals with WS exhibit the full ‘classic deletion,’ there are rare cases (∼2%) where relatively smaller deletions occur [10]. It is currently unknown how smaller WS deletions impact brain structure in WS. By investigating AWSdel, new insights into the role of specific genes on brain and behavior can be obtained. To accomplish this goal, we collected brain imaging data and behavioral data from samples of AWSdel, WS and TD. There were three types of deletions among the AWSdel cases; i.e., one where the genes *GTF2I and GTF2IRD1* were spared, another where the region from *TRIM50/FKBP6* to (but not including) *STX1A* was spared, and a third where small deletions occurred between *ABHD11* through *RFC2* including *LIMK1* (**Figure 1**). We focused on visuo-spatial and social cognition, two key phenotypes of WS and examined whether each AWSdel case resembled WS or TD. The overarching objective of this investigation was to deduce gene-brain-behavior associations by examining genes that are commonly deleted in those with similar neuroanatomical and behavioral profiles among individuals comprising our AWSdel sample (**Figure 1**).

## Material and Methods

### Ethics Statement

All procedures were approved by the Institutional Review Boards of Stanford University and Salk Institute. All participants provided written informed consent or assent. Parents or guardians provided written consent in addition to written assent if the participants were minors.

### Subjects

Participants included a total of 72 with WS, 54 with TD, and 8 individuals with AWSdel (**Table 1** **,** **2**). The 8 individuals with AWSdel had previously received a clinical diagnosis of WS. In each case, one or more genes commonly deleted in WS were spared (AWSdel-01 [ID#: 5623; spared for *GTF2I and GTF2IRD1*], AWSdel-02 [ID#: 5657; spared from *FKBP6/FZD9* up to *WBSCR22*, deletion includes *STX1A*], AWSdel-03 family i-vi [ID#s: 5882, 5884-5888; small deletions from *ABHD11* through *RFC2* including *LIMK1*) on chromosome 7q11.23 (**Figure 1**).

**Table 1:** Demographic information. WS and TD groups.

	1.5T Data (matching AWSdel-01 & AWS-del02 data)						3.0T Data (matching AWSdel-03i-vi data)					
	WS (N = 42)		TD (N = 40)		T/Chi-square tests		WS (N = 30)		TD (N = 14)		T/Chi-square tests	
	mean	SD	mean	SD	T/chi	P	mean	SD	mean	SD	T/chi	P
Age	29.20	9.00	27.47	7.36	0.95	0.35	30.01	10.23	30.77	10.00	0.21	0.65
Gender	M 19 F 23		M 16 F 24		0.07	0.79	M 13 F 17		M 7 F 7		0.01	0.92
Full-Scale IQ (ss)	68.55	8.34	104.25	12.42	12.51	<0.001	63.61	11.35	116.17	13.76	12.60	<0.001
Verbal IQ (ss)	71.90	7.43	103.31	11.03	12.37	<0.001	63.14	11.00	113.25	14.39	12.02	<0.001
Performance IQ (ss)	67.55	8.55	104.56	12.72	12.65	<0.001	68.25	11.13	115.08	11.98	11.93	<0.001
Beery VMI (ss)	49.81	7.86	91.07	9.91	15.92	<0.001	51.50	8.03				
Benton JLO (crpr)	11.54	6.64	26.18	2.35	12.36	<0.001	9.92	7.22				
Block Design (ss)	4.32	1.68	12.25	2.32	14.11	<0.001	3.34	1.80				
Approachability (raw)	17.62	7.77	7.81	7.47	4.34	<0.001	15.04	6.59				
Lt Amygdala Volume (ml)	1.80	0.34	1.60	0.33	2.72	0.008	1.85	0.34	2.10	0.28	2.53	0.02
Rt Amygdala Volume (ml)	1.65	0.26	1.46	0.29	3.12	0.003	1.77	0.32	1.88	0.25	1.22	0.23

See also Figure 2B.

F: female, M: male, VMI: Visuo-Motor Integration, JLO: Judgement of Line Orientation, ss: standard score, crps: raw score adjusted for age and gender, raw: non-normed raw score. Amygdala gray matter volumes adjusted for total cranium gray matter volumes.

**Table 2:** Demographic information. Atypical deletion cases and their deviations from WS and TD groups.

	AWSdel-01			AWSdel-02			AWSdel-03i			AWSdel-03ii	AWSdel-03iii	AWSdel-03iv	AWSdel-03v	AWSdel-03vi
	mean	vs. WS (Z)	vs. TD (Z)	mean	vs. WS (Z)	vs. TD (Z)	mean	vs. WS (Z)	vs. TD (Z)					
**Age**	41.0	1.31	**1.84**	29.9	0.08	0.33	38.8	1.06	1.54	15.8	14.6	12.9	10.6	9.4
**Gender**	M			M			M			F	F	F	F	M
**Verbal IQ (ss)**	90	**2.44**	-1.21	84	1.63	**−1.75**	88	**2.17**	**−**1.39	74	74	84	76	85
**Performance IQ (ss)**	87	**2.27**	**−**1.38	78	1.22	**−2.09**	82	**1.69**	**−1.77**	80	73	94	81	75
**Full-Scale IQ (ss)**	88	**2.33**	**−**1.31	79	1.25	**−2.03**	84	**1.85**	**−**1.63	75	71	87	77	78
**Beery VMI (ss)**	75	**3.21**	**−**1.62	54	0.53	**−3.74**	81	**3.97**	**−**1.02	88	72	93	83	80
**Benton JLO (crpr)**	25	**2.03**	**−**0.50	15	0.52	**−4.75**	17	0.82	**−3.90**					
**Block Design (ss)**	9	**2.79**	**−**1.40	10	**3.39**	**−**0.97	7	1.60	**−2.26**					
**Approachability (raw)**	21	0.44	**1.77**	26	1.08	**2.44**	28	1.34	**2.70**					
**Lt Amygdala Volume - adjusted (ml)**	2.26	1.34	**1.98**	2.27	1.36	**2.01**	2.33	1.55	**2.21**					
**Rt Amygdala Volume - adjusted (ml)**	1.67	0.08	0.72	2.19	**2.08**	**2.51**	1.77	0.46	1.06					

See also Figure 2B.

F: female, M: male, VMI: Visuo-Motor Integration, JLO: Judgement of Line Orientation, ss: standard score, crps: raw score adjusted for age and gender, raw: non-normed raw score. Amygdala gray matter volumes adjusted for total cranium gray matter volumes. Bold values are those that are significant.

Exclusion criteria included a history of significant medical and neurological conditions or symptoms not known to be associated with WS such as cerebral palsy, stroke, multiple sclerosis, Parkinson's disease or head trauma resulting in loss of consciousness. Epilepsy occurs in WS but does not have as high a rate as in other neurodevelopment conditions such as autism, fragile X and tuberous sclerosis. Thus, these were exclusions for WS (typical AND atypical) as well as for controls. All WS and AWSdel participants received cognitive-behavioral, genetic and imaging assessments as part of a multi-site program project grant on genetics, neuroanatomy, neurophysiology, and cognition.

Healthy control participants (with no history of major psychiatric, neurological, or cognitive impairment) were recruited at both the Salk Institute and Stanford University. TD control participants were further screened to rule out any history of learning, language, or behavioral disorder over the phone using a screening form as in our prior studies of WS and other neurogenetic conditions (e.g. [25]).

### Genetic Testing

In order to confirm the extent of each participant's deletion (typical WS and AWSdel), a series of genetic analyses were performed. Specifically, deletions in all the typical WS were confirmed by the use of Fluorescence in Situ Hybridization (FISH) with bacterial artificial chromosomes (BACs) including in all cases, probes for ELN and in addition, subsets of probes marking the ends of the typically deleted region (1008H17 for FKBP6, FZD9; 592D8 for ELN, LIMK1; and 1184P14 for GTF2I), for the typically non-deleted single copy gene, CALN1 (815K3) located upstream of the centromeric duplicated region. Probes for the duplicated regions flanking the common deletion were employed along with ELN as a screen for the common deletion [16]. For atypical participants AWSdel-01 and AWSdel-02, further FISH analyses used a total of twenty-one sequenced linked DNAs isolated from either BACs [16] or cosmids [17] to cover the region defining the deletion breakpoints as described in [18]. Quantitative PCR was performed using probes spanning from CALN1 to WBSCR16 and template DNA isolated from blood and/or lymphoblast cell lines, to determine the deleted region of atypical participants AWSdel-01, AWSdel-02, AWSdel-03i-vi [18]. Finally, a custom high resolution genomic NimbleGen array [15] spanning a 13 Mb region that included the typical deletion, flanking repeats and surrounding single copy DNA, was used to define the deletion structures in atypical participants AWSdel-01, AWSdel-02, AWSdel-03-i, iv, v [15], [19]. A schematic diagram showing atypical deletions of these 8 cases is presented in **Figure 1** **.**

### Cognitive/Behavioral Testing

Participants were given a standard battery of measures that included the Wechsler Adult Intelligence Scale (WAIS) [20] and Wechsler Intelligence Scale for Children (WISC) [21] to measure verbal, performance and full-scale IQ. IQ was available for all but 5 WS and 2 TD. There were no significant differences between the participants with and without IQ in level of cognitive function on any measure listed in **Table 1** (all p's >0.1).

Participants also completed a series of tasks designed to assess visuo-spatial functioning. This included the Block Design subtest of the IQ tests (a timed test where the participant arranges the blocks with white and/or red sides according to a pattern), Beery Visuo-Motor Integration [22] (the participant reproduces simple line drawings) and non-normed Benton Judgment of Line Orientation test [23] (the participant identifies the slope of the presented lines on the display of 11 lines).

Lastly we used a non-normed test to measure social approachability in each participant [24]. This task involves participants reporting how likely they would approach a person depicted in a photograph.

WS individuals showed significantly reduced IQ and visuo-spatial abilities, and significantly higher social approachability scores (all p's<0.05). Please see **Table 2** for mean scores, statistics, and scores of partial deletion cases. Analyses of cognitive profiles were performed in parallel to the anatomical brain measures described below with a cut-off score of z = 1.65 (1-tailed) instead of z = 1.96 that was used for brain measures.

### Acquisition of Anatomical Brain Measures

MRI data were collected on two scanners (1.5 T and 3.0 T, respectively). Data from 2 of the AWSdel (AWSdel-01, AWSdel02), 42 WS and 40 TD individuals were obtained using a 1.5T MRI scanner. Data from AWSdel-03i ∼ AWSdel03vi (father and 5 adolescent children), 30 WS and 14 TD individuals were obtained at a subsequent time using a 3.0T MRI scanner. The **Main Text** reports findings comparing AWSdel (including AWSdel-03 data obtained from a 3.0T) to WS and TD data from 1.5T. The results were unchanged when 3.0T reference data from individuals with WS and TD MRI served as the reference to which MRI data from individuals with AWS (acquired from both 1.5T and 3.0T scanners) were compared (see **Supporting Results**).

All 1.5T data (42 WS, 40 TD, AWSdel-01, AWSdel-02) were acquired with a GE-Signa 1.5T scanner (General Electric, Milwaukee, WI) located at one of three sites: University of California, San Diego Medical Center Magnetic Resonance Imaging Institute (N = 59), Scripps Clinic, San Diego (N = 53), or Stanford University (N = 5). Across all locations and in all cases, sagittal brain images were acquired with the same three-dimensional (3D) volumetric radio frequency spoiled gradient echo (spoiled gradient-recalled acquisition in a steady state) pulse sequence using the following scan parameters: repetition time, 24 msec; echo time, 5 msec; flip angle, 45°; number of excitations, 2; matrix size, 256 × 192; field of view, 24 cm; slice thickness, 1.2 mm; 124 contiguous slices.

The remaining participants (30 WS, 14 TD, AWSdel-03i∼vi) underwent MRI on a GE-Signa 3.0 T scanner (General Electric, Milwaukee, WI) at Stanford University. In these cases, coronal brain images were acquired with a three-dimensional enhanced fast gradient echo (EFGRE3D) pulse sequence using the following scan parameters: repetition time, 6 msec; echo time, 1.5 msec; flip angle, 15°; number of excitations, 3; matrix size, 256 × 256; field of view, 24 cm; slice thickness, 1.5 mm; 124 contiguous slices.

### Voxel Based Morphometry (VBM) Processing

VBM analyses of 42 WS and 40 TD MR images from the 1.5T scanners were performed using SPM5 (http://www.fil.ion.ucl.ac.uk/spm) and VBM5.1 (http://dbm.neuro.uni-jena.de/vbm). After bias correction, T1 images were segmented into gray matter, white matter and cerebral spinal fluid. Hidden Markov Random Field (prior probability weight 0.3) was used to encode spatial information through spatial constraints of neighboring voxels. Normalization was performed using both adult and custom templates created from the 42 individuals with WS and 40 with TD. Both Jacobian modulated (non-linear warping only; reflecting regional gray matter volume) and nonmodulated (reflecting gray matter density) images were smoothed with an isotropic Gaussian kernel with full-width at half-maximum (FWHM) of 8 mm. Analyses of gray matter density were performed to compare with our previous report on WS individuals [25]. Segmentation and normalization for each participant was confirmed by manual inspection of the images. These steps were repeated for the 30 WS and 14 TD MR images collected using 3.0T.

### Regions of Interest (ROIs)

ROIs were restricted to brain regions that showed significant differences between WS and TD individuals in gray matter images processed using VBM (**Figure S1, Table S1**). Results comparing WS and TD are reported in **Supporting Results** and not in the **Main Text** because the results (from 1.5T) have been reported previously (e.g. [25]) and results from 3.0T are used to replicate our findings. Our particular interest was in the intraparietal sulcus (IPS; right more than left), thought to be critical for visuo-spatial processing, and the amgydala, orbitofrontal cortex and fusiform gyri (right more than left) thought to be critical for social cognition and face processing. While the amygdala did not reach significance comparing WS and TD with VBM, based on evidence that the amygdala is structurally and functionally abnormal in WS and its importance in socio-emotional functioning [25], [26], this region was manually delineated as described below.

### Volumetric Measures of the Amygdala

Amygdala volumes were obtained from delineation based on anatomical landmarks by trained research staff who followed a detailed protocol [25]. Briefly, the amygdala delineation was initiated on the coronal slice where the anterior commissure was best distinguished and proceeded in the posterior direction until both the amygdala and hippocampus were clearly visible on the same slice. Superior, inferior and lateral boundaries were each designated by prominent white matter tracks, while the medial boundary was designated by either white matter or cerebral spinal fluid. Inter-rater reliabilities for all volumes described in this study were ≥ 0.90 as determined by the intraclass correlation coefficient.

### Univariate Analyses

Brain images of each AWSdel case processed using VBM were compared to the WS and TD datasets (see **Supporting Methods**). We generated two voxel-wise z-score maps per AWSdel case that indicated how each AWSdel's brain image deviated from WS and TD groups (thresholded at z>1.96 and z<−1.96, p's<0.05). For example, if a partial deletion case showed |z|>1.96 from one group and |z|<1.96 from the other, then it was determined that the partial deletion case resembled the latter group. In the case of the AWSdel-03 family (father and 5 children with same atypical WS deletion), after creating these thresholded z-score maps for each family member AWSdel-03i∼vi, we further created probabilistic maps using the thresholded images to examine the proportion of family members that showed z>1.96 or z<−1.96. These z-score and probabilistic images allowed us to visualize whether each AWSdel case showed a relative propensity for WS-like versus TD-like neuroanatomical patterns.

Results comparing WS and TD groups utilizing standard SPM5 and custom templates (**Figure S1, Table S1**), gray matter volume and density (**Figure S1**), and 1.5T and 3.0T scanners (**Figure S2**) are also reported in **Supporting Results**. AWSdel results comparing standard gray matter volume and density (**Figure S3**) and 1.5T and 3.0T scanners (**Figure S4**), are also reported in **Supporting Results**. There were no observable differences in results obtained from different templates, measures (volume vs. density) and magnet strength that appeared to influence the current findings. Further, though age and gender were corrected for all imaging analyses, the results did not change; hence we report findings without these covariates.

### Multivariate Pattern Classification Analyses (MVPA)

Cross-validated linear support vector machine (SVM) was utilized as the primary method of performing MVPA to accomplish two objectives. First, these analyses were conducted as a complementary approach to univariate analyses described above for investigating whether voxels in brain regions related to visuo-spatial functioning and social cognition would classify each AWSdel individual as belonging to either the WS or TD group. Second, MVPA was used to investigate whether each AWSdel individual would be classified as WS or TD when the entire population of either gray matter or white matter voxels were considered.

Voxels included in the MVPA were segmented using one of two approaches: 1) bilateral superior parietal lobules (SPL), orbitofrontal cortices (OFC), amygdalae and fusiform gyri were defined using the WFU PickAtlas (http://fmri.wfubmc.edu/cms/software#PickAtlas) and Automated Talairach Atlas Label (AAL) (http://www.cyceron.fr/freeware/), then coregistered to the modulated VBM gray matter images, and 2) masks comprising all brain gray or white matter voxels were obtained from the SPM5 generated segmentation. Non-smoothed images were down-sampled to 4mm voxels and voxel-by-subject matrices were created for each (set) of these brain regions. Linear support vector machine (SVM) analyses were performed (regularization parameter C = 1), with leave-one-out cross-validation using an in-house toolbox used in previous studies [27]–[33]. Feature reduction was performed using leave-one-out recursive feature elimination (RFE), recursively eliminating 30% of the voxels to identify the optimal performance [34], [35]. A classifier from each of the leave-one-out cross-validation steps was applied to each AWSdel individual, allowing us to calculate the probability of each AWSdel individual being classified as an individual with WS or TD. As reference, visuo-spatial regions classified WS from TD at 100% accuracy, social regions at 95.1% accuracy, gray matter at 97.6% accuracy and white matter at 98.8% accuracy. These references indicate that MVPA is capable of classifying WS and TD controls and serves as a basis for performing further MVPA on AWSdel cases.

## Results

### Neuroanatomical and Behavioral Abnormalities Associated with Visuo-Spatial Function in Atypical WS Deletion Cases (AWSdel)

**Figure 2:**
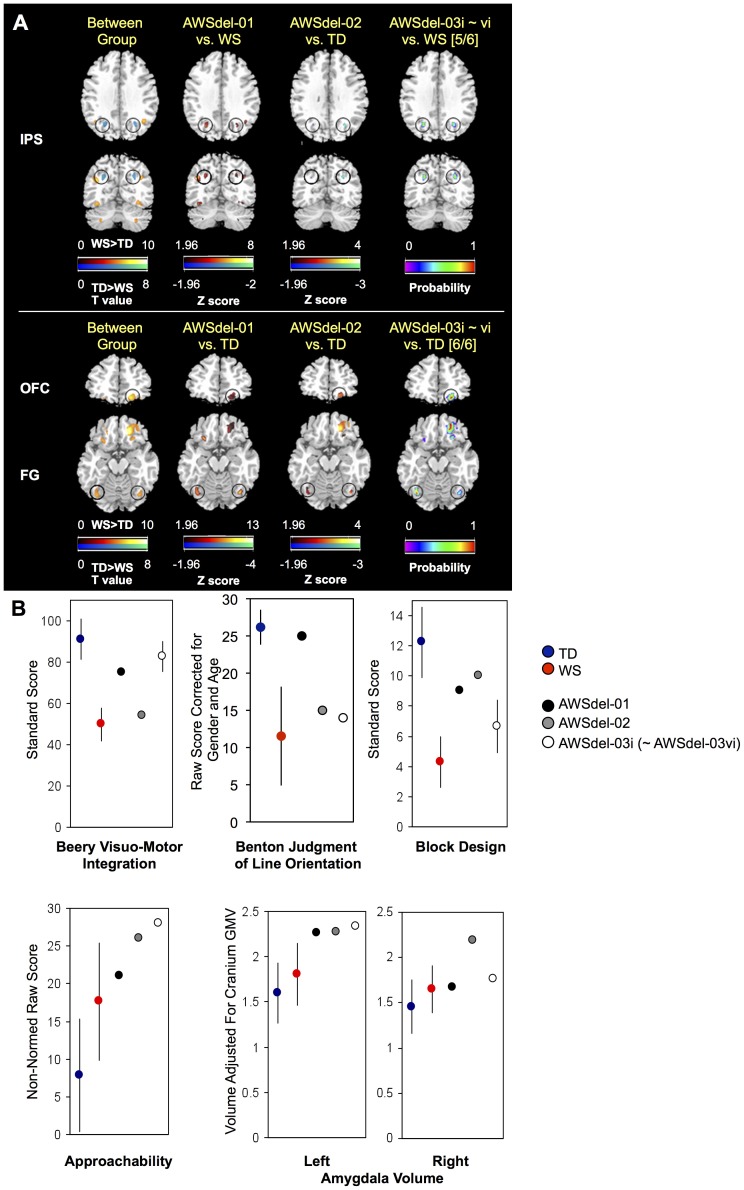
Gray matter volumes and cognitive profiles of typical WS, TD, and atypical deletion (AWSdel) individuals. **A.** Gray matter deviation maps in AWSdel individuals. First column represents VBM between group differences between WS (N = 42) and TD (N = 40). Second and third columns represent the degree to which atypical cases AWSdel-01 and AWSdel-02 deviated from the comparison group (thresholded at [z]>1.96). The fourth column represents probability maps of how many participants showed positive deviation of z > 1.96 in AWSdel-03i∼vi. Numbers in square-brackets in the fourth column indicate how many participants out of the total of 6 AWSdel-03 participants showed this deviation in its peak voxel. **B.** Cognitive measures and amygdala volumes (from manual volumetric measurements) are plotted for WS, TD and AWSdel groups. See **Table 1** for detailed statistics. Benton judgment of line and Social approachability scores are not plotted for the AWSdel-03 children (WSdel-03ii∼vi) as age-adjusted normed scores are not available. IPS: intraparietal sulcus, OFC: orbitofrontal cortex, FG: fusiform gyrus, Lt: left, Rt: right. Error bars represent standard deviation. Left hemisphere is shown on the left side in the brain maps.

Results from the IPS, a region that plays a central role in visuo-spatial processing [36] and in which a specific cluster was defined from the between-group WS vs. TD comparison (see **Supporting Results, Figure S1**, and **Figure S2**), are shown in **Figure 2A** **and** **Table 3** (see also **Figure S3, Figure S4**). AWSdel-01 showed voxels with values exceeding z>1.96 compared to WS, but no voxels with values that were |z|>1.96 when compared to TD, indicating that AWSdel-01 shows IPS volumes within the distribution of TD. In AWSdel-02, voxels with values z<−1.96 compared to TD were found in the IPS, but no voxels with values that were |z|>1.96 when compared to WS, indicating that AWSdel-02 shows IPS volumes comparable to WS. Further, in AWSdel-03, all family members showed voxels with values within the range of TD (|z|<1.96) and greater than WS (all members showed z>1.96), indicating that AWSdel-03 shows IPS volumes similar to TD. Since MRI data of the AWSdel-03 family were collected from 3.0T rather than the 1.5T reference data from 42 WS and 40 TD participants, AWSdel-03 cases were also compared to the 3.0T reference dataset of 30 WS and 14 TD participants. Results from this 3.0T scanner comparison (**Figure S4**) were essentially identical to the 1.5T results in **Figure 2A**.

**Table 3:** Z-scores of each WS atypical deletion case (AWSdel) from the WS or TD group in each brain region of interest: Left/right (Lt/Rt intraparietal sulcus (IPS), Rt orbitofrontal cortex (OFC), and Lt/Rt fusiform gyrus (FG).

	Lt IPS	Rt IPS	Rt OFC	Lt FG	Rt FG
AWSdel-01	6.00	5.28	7.79	8.02	9.74
AWSdel-02	−2.03	−2.65	2.15	3.20	2.26
AWSdel-03	3.41(2.12)	2.86(2.09)	3.28(1.36)	4.18(2.43)	2.55(0.77)

Numbers indicate # of Z scores above the TD group with the exception of AWSdel-02 Lt/Rt IPS which indicate Z scores below the WS group. When compared to WS, Z<1.96 except AWSdel-02 Rt Amyg was Z = 2.08 compared to the WS group. AWSdel-03 family (03i ∼ 03vi) are listed as the mean average of the 6 members and standard deviation in brackets.

We created a classifier (model) designed to optimally discriminate between WS and TD using permutation-based MVPA. MVPA was performed using voxel intensity measures from objectively and anatomically defined left and right superior parietal lobules (SPL) within VBM processed images. Consistent with VBM results, using permutation-based analyses, AWSdel-01 and all AWSdel-03 family members were categorized as TD with 100% probability, and AWSdel-02 was categorized as WS with 100% probability.

**Figure 3:**
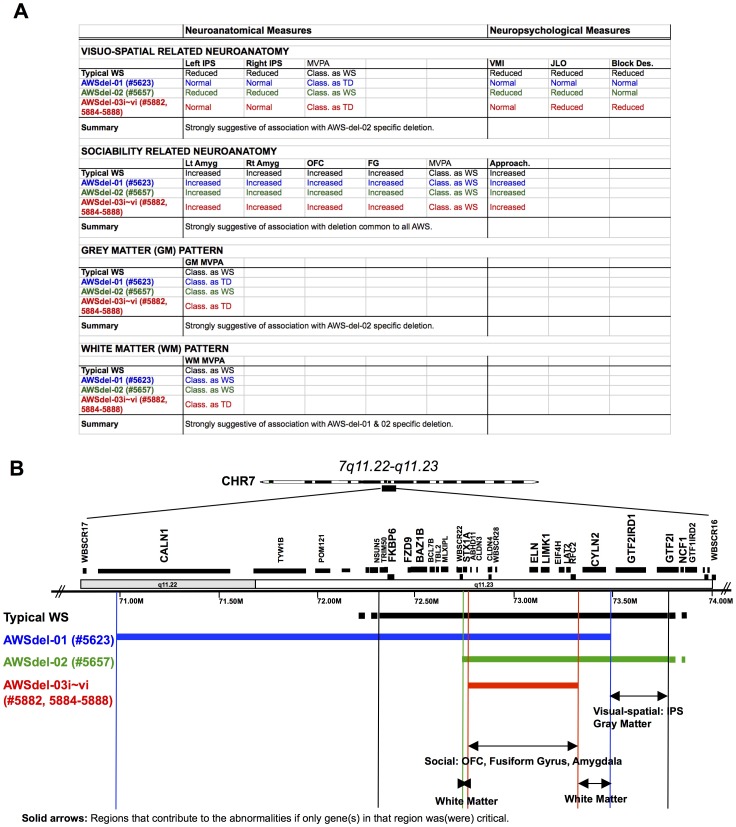
Schematic table (A) and diagram (B) that represent summary of findings.

Behavioral performance on visuo-spatial functions (Beery Visuo-Motor Integration, Benton Judgment of Line Orientation and Wechsler Block Design tasks) for each AWSdel case compared to that of WS and TD individuals generally paralleled the neuroanatomical results (**Table 1** **,** **2** **,** **Figure 2B**). The results of this analysis showed that AWSdel-01 was within or closer to (for visuo-motor integration and block design) the range of TD, AWSdel-02 was within the range of WS (clearly for visuo-motor integration and judgment of line orientation, though not for block design), and AWSdel-03 was within the range of TD (though in this case only for visuo-motor integration and not for judgment of line orientation or block design). Overall, these results implicate *GTF2I* and/or *GTF2IRD1* as candidate genes contributing to altered IPS volumes and visuo-spatial function, in particular visuo-motor integration as consistent with previous animal [37] and human behavioral research [15], [38] (**Figure 3**).

### Neuroanatomical and Behavioral Abnormalities Associated with Social Cognitive Function in Atypical WS Deletion Cases (AWSdel)

Next, we examined morphometric patterns related to social cognitive function in AWSdel. All AWSdel cases showed increased volumes in bilateral fusiform gyri and the right orbitofrontal cortex (OFC) compared to TD (z>1.96); this profile was similar to typical WS participants (|z|<1.96) (**Figure 2A** **,** **Table 3** **, Figure S3, Figure S4)**. In the manually delineated amygdala, we found that WS showed significantly greater gray matter volume in bilateral amygdala compared to TD (**Table 1**). In the left amygdala, all AWSdel cases showed increased volume compared to TD (z>1.96), which was similar to WS (|z|<1.96) (**Figure 2B**). Using permutation-based MVPA, we observed that a combination of objectively defined amygdala, OFC and fusiform gyrus regions of interest showed 100% probability (using permutation-based analysis) that all AWSdel cases would be categorized as WS.

Subject performance on social behavior (Adolph's social approachability test) for each AWSdel case was compared to average social behavior scores in WS and TD groups. The results of this analysis showed that all AWSdel cases exhibited increased sociability relative to TD controls, again paralleling the neuroanatomical findings (**Table 2** **,** **Figure 2B**). Each case was more similar to the WS group relative to the TD group. There are several genes commonly deleted in our typical WS and AWSdel groups (genes located between *ABHD11* and *RFC2*). However, one gene in particular, *LIMK1*, is known to affect brain development [39], thus suggesting that haploinsufficiency of *LIMK1* as one potential explanation for aberrant OFC, fusiform and amygdala volumes, as well as abnormalities of social approachability in WS (**Figure 3**).

### Neuroanatomical Abnormalities of Overall Gray and White Matter Patterns in Atypical WS Deletion Cases (AWSdel)

In our final analyses, we applied cross-validated MVPA to investigate gene(s) that potentially contribute to distinct patterns as derived from voxel-by-voxel volumes comprising the entire gray matter and white matter compartments. Using permutation-based analyses, gray matter results showed that, similar to visuo-spatial processing, AWSdel-01 was categorized as TD with 89.0% probability (and hence as WS with 11% probability), AWSdel-02 as WS with 100% probability and all AWSdel-03 family members as TD with 100% probability. White matter results indicated that AWSdel-01 was categorized as WS with 98.9% probability, AWSdel-02 as WS with 100% probability and AWSdel-03 family members as TD with 100% probability. These results suggest that gray matter structure varies with the presence or absence of *GTF2I/GTF2IRD1*, while white matter structure is most related to the status of genes in the region of *STX1A* and *CYLN2* which are common to AWSdel-01 and AWSdel-02 but outside the deletion in AWSdel-03 (**Figure 3**). Deleted genes in WS that are particularly important in the regulation of cytoskeletal dynamics and possibly in white matter development are *LIMK1*, *STX1A*, *CYLN2 and* possibly *FZD9* [4], [40]. Among these genes, *STX1A* and *CYLN2* are deleted in AWSdel-01 and AWSdel-02 and are spared in AWSdel-03 raising the possibility that *STX1A* and *CYLN2* may be among the genes involved in white matter development in WS.

## Discussion

Our results provide evidence implicating particular genes in the development of brain structures involved in visuo-spatial function and social cognition in humans. If the contribution of single genes is measureable and related to the characteristic pattern that we observe in this study, the findings indicate a role for *GTF2I* and/or *GTF2IRD1* in IPS volumes known to be involved in visuo-spatial function, and more generally, in patterns of gray matter structure; the *LIMK1* region (and possibly other genes located between *ABHD11* through *RFC2*) in the volumes of the amygdala, OFC and fusiform gyrus, which are known to be involved in social approachability; and *CYLN2/STX1A* in white matter development.

The right IPS has been consistently shown to be involved in visuo-spatial constructions, including visuo-motor integration [36]. Evidence suggests that visuo-motor deficits may be one of the most characteristic features of WS [41], and the IPS results in our 8 atypical cases (with 3 different patterns of atypical deletions) point to *GTF2I* and/or *GTF2IRD1* as critical for the performance of these tasks, as suggested previously [15], [38]. The way by which these genes affect the anatomy and behavior demonstrated in this study is not known. *GTF2I* encodes a multifunctional phosphoprotein with roles in transcription and signal transduction [42]. The protein encoded by *GTF2IRD1* contains five GTF2I-like repeats and functions as a transcription factor or as a positive transcriptional regulator under the control of the Retinoblastoma protein. Both *Gtf2i* and *Gtf2ird1* are widely expressed in the brain during the embryonic stages of mouse development [43]. In adult mice, they are present exclusively in neurons, but the two proteins play nonredundant, differentially regulated roles, despite their similar structure. At present, the specific genes whose expression are most affected by these regulatory genes are not known. Nor is it known how, and to what extent, *Gtf2i* and *Gtf2ird1* are explicitly involved in IPS morphometric variability, although converging evidence from our previous behavioral study [15] suggests *GTF2IRD1* to be principally responsible.

The cognitive profiles examined here, though partially consistent with the anatomical findings, showed some discrepancies. Visuo-motor integration was consistent with IPS volume findings throughout all cases, but judgment of line orientation and block design results were inconsistent in the AWSdel-03 family (i.e., judgment of line orientation and block design showed more WS-like patterns even though IPS and visuo-motor integration showed TD-like patterns). However, this is not altogether surprising, since behavior is downstream to genetics and neuroanatomy, and hence may be influenced in many different ways that are difficult to reliably assess (as opposed to the more upstream neuroanatomical measure) [1]. Alternatively, it may be that in WS, *GTF2I/GTF2IRD1* have greater involvement in IPS and visual construction and motor components (represented by visuo-motor integration) than visual perceptual functions (represented by judgment of line orientation and block design). In addition, multiple WS genes could contribute to decreased visual perceptual functions.

There is limited behavioral research on WS with partial deletions [11]–[15]. The current findings replicate our previous report showing the important role of *GTF2IRD1* in visuo-spatial abilities [15]. However, our study results contradict other behavioral studies of visuo-spatial abilities [11]–[15]. Karmiloff-Smith et al. [11] reported an atypical deletion case with a deletion from *FKBP6* to *GTF2IRDI* and showed this case to be associated with relatively spared visual-spatial functioning and abnormal social behavior. Antonell et al. [13] reported that the deletion of *GTF2I* and *GTF2IRD1* may contribute to the global intellectual deficit and some aspects of the cognitive profile but not visuo-spatial ability. Tassabehji et al. [12] and Gray et al. [14] showed an association between *LIMK1* and visual-spatial construction.

One of the most striking features of WS is gregariousness, reduced fear for strangers, and relative preservation of a subset of face processing skills. The amygdala is known to be an important component of the neural systems involved in retrieving socially relevant knowledge on the basis of facial features, in particular approachability and trustworthiness [24], probably through its role in regulating fear. Further, the OFC is known to regulate amygdala reactivity through a frontal-amygdala-insular circuit that provides feedback about somatic state activation, which aids in social decision-making [44], [45] and empathy [46], [47]. Finally, the (right) fusiform gyrus is considered to be a face processing region that is particularly important for normal social interactions, and provides input to the anterior limbic regions [48]. Typical WS and all AWSdel participants in this study who have deletions of genes from *ABHD11* through *RFC2* including *LIMK1*, showed developmental anomalies of the amygdala, OFC and/or fusiform cortex. *LIMK1* is a particularly a strong candidate among the deleted genes for playing a role in the anomalous neuroanatomical pattern described above because of its known role in brain function and dendritic spine architecture [39],

*LIMK1* is thought to be a component of an intracellular signaling pathway involved in brain development. In particular, *LIMK1* controls actin dynamics via phosphorylation of cofilin, and has been implicated in the control of growth cone motility in cultured neurons [49] and in white matter development [50]. It is difficult to disentangle from our study which neurobiological pathways or neural structures are more directly influenced by *LIMK1* and which brain regions are affected by a combination of interactions among genes in the deleted regions. Nevertheless, it is attractive to propose *LIMK1*, as one gene contributing to abnormal social cognitive profiles in WS, either via impaired input from higher-level visual (fusiform face) areas or through interactions between the OFC and amygdala. It is important to note that these effects may derive from both pre- and post-natal effects on brain development or adult function. Future studies examining the causal relationship of brain activation patterns within these structures in the context of social-affective processing will be of interest.

There are several genes that have been implicated in WS social cognition. Although *LIMK1* was an attractive target, there is only one animal study to date that has suggested a role for *LIMK1* in social cognition (fear responses) and this finding was interpreted within the context of impaired learning and hippocampal function [39]. In other studies, *LIMK1* has only been implicated in visuo-spatial function [51]. In contrast, recent literature on social cognition in WS points to a potential role for *GTF2I/GTF2IRD1* [15], [43], [52], regions between *LIMK1* and *GTF2I/GTF2IRD1* [53], or regions between *FKBP6* and *GTF2IRD1* [11]. These genes are further implicated by mice with heterozygous or homozygous disruption of *Gtf2ird1* who exhibit decreased fear, aggression and anxiety and increased social behaviors as well as increased levels of serotonin metabolites in the amygdala and frontal cortex [43]. Future studies using multiple standardized and non-standardized behavioral measures, in larger samples in conjunction with heterozygous animal models, which can examine single genes at a time, and examining both brain structure and cognitive and social profiles, are warranted.

Finally, *LIMK1*, *CYLN2* and possibly *FZD9* have been implicated in WM development. For example, *LIMK1* and *CYLN2* are known to regulate cytoskeletal dynamics, axon guidance and neuronal migration, and *FZD9* may be critical for dendritic development and axon guidance [4], [40]. Our results suggested that genes, including *STX1A* and/or *CYLN2*, may contribute to WM development in WS.

Mice with heterozygous and homozygous disruptions of particular genes are potentially ideal models to study gene-brain-behavior associations, but the behavioral tests available can be limited depending on the behavior/cognitive function of interest. Further, the same genes in humans may not have the same regulatory pathways and expression patterns as that occurring in the brains of non-humans [11]. Some animal studies showing statistical differences between wild-type and homozygous knockout mice fail to demonstrate comparable differences when using heterozygous mice, the genetic state most applicable to WS. Further, the scarcity of AWSdel cases have resulted in only one or two cases described in each report [11]–[15]. These factors have made it difficult to attribute specific cognitive functions to particular sets of genes. Our study attempted to overcome some of these limitations by examining eight individuals with three types of varying atypical deletions, while also assessing brain structure for the first time in AWSdel cases.

A major limitation is that our current level of analysis only allows attribution of subsets of genes to neuroanatomical and cognitive findings. It is likely that there is more than one contributory gene, gene-gene interactions within and outside the WS deletion, as well as environmental influences and stochastic processes that could contribute to neuroanatomical variations. Another limitation is that, similar to typical controls, individuals with WS demonstrate inter-individual differences in neuropsychological and behavioral function and hence future studies employing more sensitive experimental behavioral measures are warranted. Further, while this is the first study to utilize AWSdel to examine gene-brain-behavior relationships, the small sample-size (8 cases with 3 patterns of deletions) will necessitate replication in future studies. Finally, in contrast to neuroanatomical volumes that are more readily quantified, pinpointing a relationship of these to behavior or cognition is limited by the extent to which a given paradigm mirrors the function of the structure. Therefore, as a first step, we have focused on demonstrating that individuals with AWSdel exhibit structural brain patterns or cognitive profiles that are either consistent or inconsistent with typical WS or TD. Future studies will be useful in refining structure-function relationships in, as well as interactions/connectivity amongst these regions; such studies can help to narrow gene candidates that alter the development or function of specific functional human brain circuits.

In summary, we show that the current preliminary study in individuals with rare, atypical deletions associated with WS provide new insights into the neural mechanisms of cognitive function and putative genetic underpinnings. These studies of intermediate endophenotypes should prompt future research into the relevance of variation in these genes, gene-gene interactions, and developmental and individual differences in gene expression, for regional brain development and normal visuo-spatial function and social behavior.

## Supporting Information

Figure S1 **Gray matter volume and density differences between WS (N = 42) and TD (N = 40) groups in 1.5T MRI data.** Either a custom template created including all WS and TD participants or a standard template provided by SPM5 was used. p = 0.05 family-wise error (FWE), extent threshold (ET) = 100.
(JPG)

Figure S2 **Gray matter volume measures from the two separate scan parameters (1.5T WS: N = 42, TD: N = 40; 3.0T WS: N = 30, TD = 14) in the regions of interest (ROIs) are plotted and compared.** See Main Text Methods for definition of ROIs and how bilateral intraparietal sulcus (IPS), right orbitofrontal cortex (OFC) and right fusiform gyrus (FG) were defined. The results are very similar between the two datasets (∼*: 0.05 < p < 0.10, **: 0.01 < p < 0.05, ***: p < 0.001).
(JPG)

Figure S3 **Gray matter density deviation maps of WS atypical deletion (AWSdel) cases.** Identical to Figure 2A, but examining gray matter density rather than volume.
(JPG)

Figure S4 **Probabilistic maps of participants AWSdel-03i∼vi (collected on a 3.0T scanner).** Identical to Figure 2A 4^th^ column (which is for gray mater volume) and Figure S3 4^th^ column, with the exception that Figure S4 uses WS and TD data from 3.0T MRI as comparison groups to match scan parameters with AWSdel-03i∼vi.
(JPG)

Table S1 **Gray matter volume differences between WS (N = 42) and TD (N = 40) groups.** A custom template was used. p = 0.05 family-wise error (FWE), extent threshold (ET) = 100.
(DOCX)

File S1 **Textual supporting information.**
(DOC)
