# Supplementary figures and images for: Mapping Genetically Controlled Neural Circuits of Social Behavior and Visuo-Motor Integration by a Preliminary Examination of Atypical Deletions with Williams Syndrome
Source: PLoS One. 2014 Aug 8;9(8):e104088. doi: 10.1371/journal.pone.0104088 (PMC4126723; doi:10.1371/journal.pone.0104088)

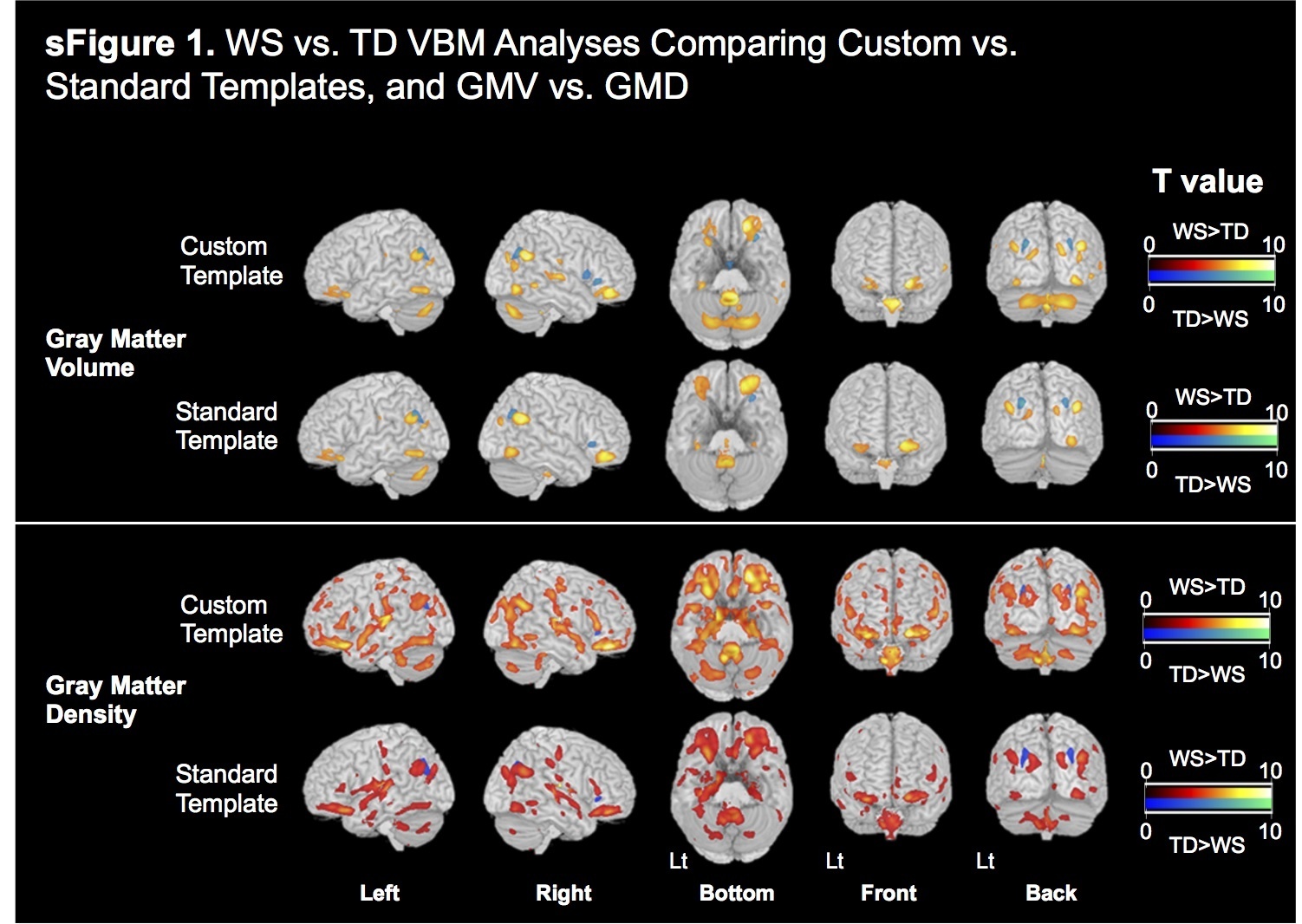

Supplement: Figure S1 — Gray matter volume and density differences between WS (N = 42) and TD (N = 40) groups in 1.5T MRI data. Either a custom template created including all WS and TD participants or a standard template provided by SPM5 was used. p = 0.05 family-wise error (FWE), extent threshold (ET) = 100. (JPG) [file pone.0104088.s001.jpg]

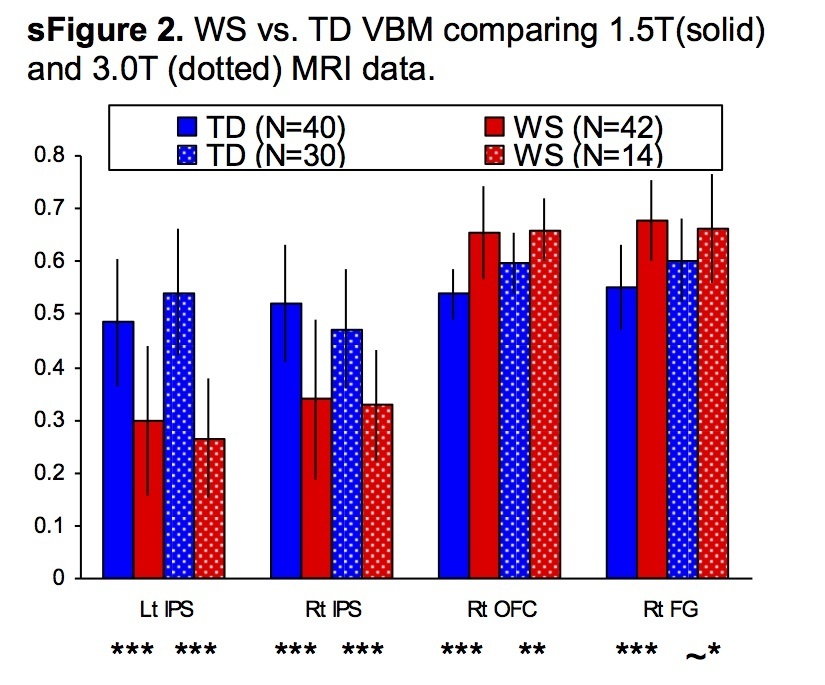

Supplement: Figure S2 — Gray matter volume measures from the two separate scan parameters (1.5T WS: N = 42, TD: N = 40; 3.0T WS: N = 30, TD = 14) in the regions of interest (ROIs) are plotted and compared. See Main Text Methods for definition of ROIs and how bilateral intraparietal sulcus (IPS), right orbitofrontal cortex (OFC) and right fusiform gyrus (FG) were defined. The results are very similar between the two datasets (∼*: 0.05 < p < 0.10, **: 0.01 < p < 0.05, ***: p < 0.001). (JPG) [file pone.0104088.s002.jpg]

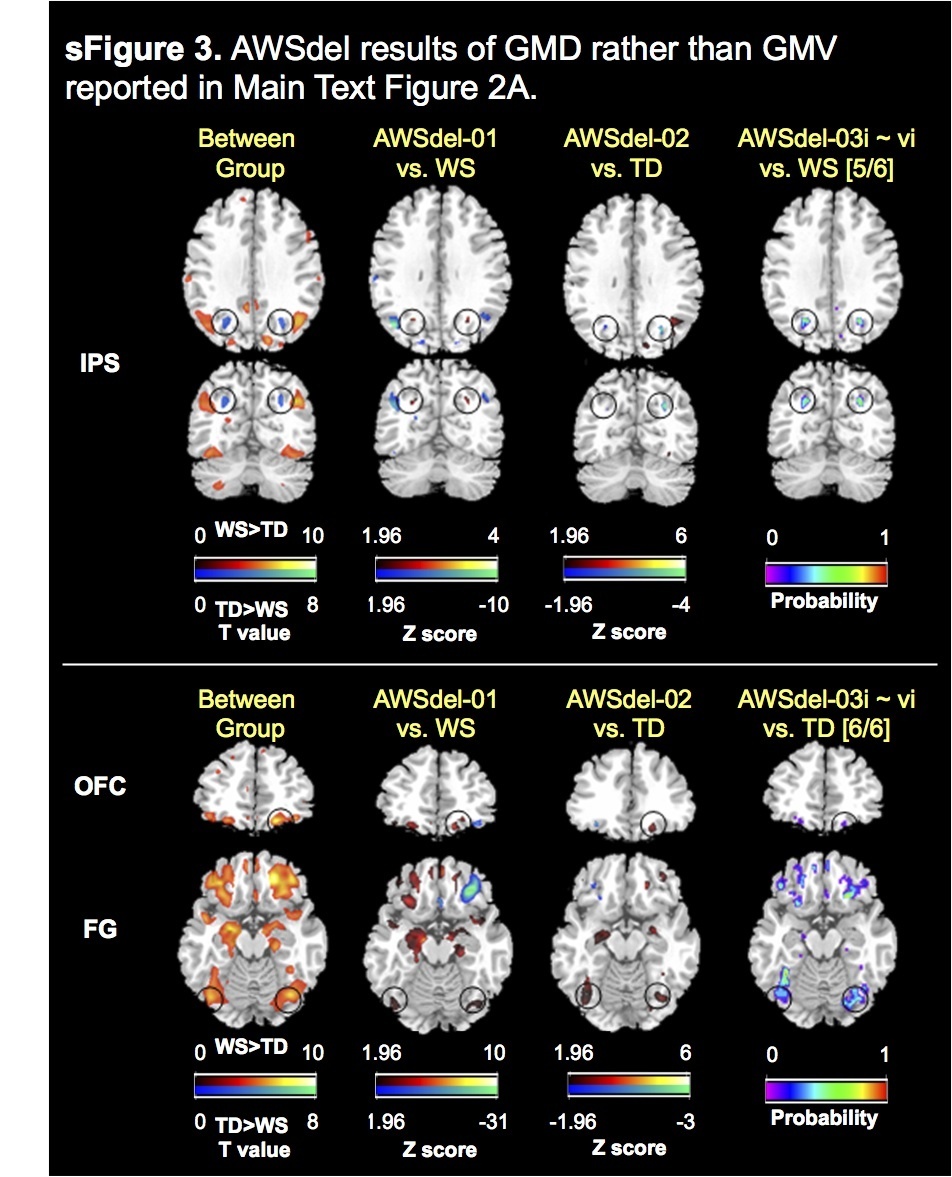

Supplement: Figure S3 — Gray matter density deviation maps of WS atypical deletion (AWSdel) cases. Identical to Figure 2A, but examining gray matter density rather than volume. (JPG) [file pone.0104088.s003.jpg]

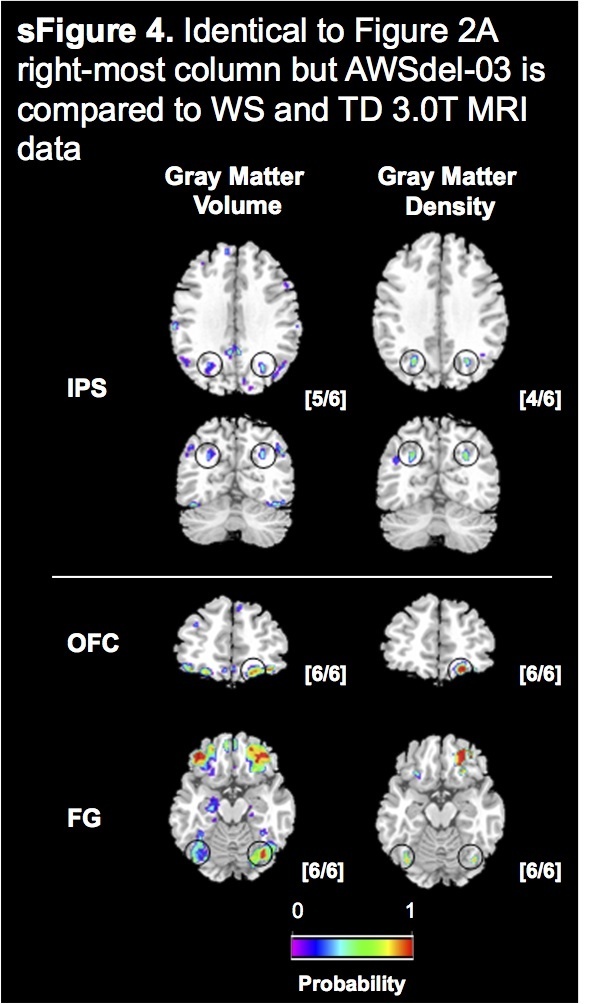

Supplement: Figure S4 — Probabilistic maps of participants AWSdel-03i∼vi (collected on a 3.0T scanner). Identical to Figure 2A 4th column (which is for gray mater volume) and Figure S3 4th column, with the exception that Figure S4 uses WS and TD data from 3.0T MRI as comparison groups to match scan parameters with AWSdel-03i∼vi. (JPG) [file pone.0104088.s004.jpg]
