# Supplementary material for: Mapping Genetically Controlled Neural Circuits of Social Behavior and Visuo-Motor Integration by a Preliminary Examination of Atypical Deletions with Williams Syndrome
Source: PLoS One. 2014 Aug 8;9(8):e104088. doi: 10.1371/journal.pone.0104088 (PMC4126723; doi:10.1371/journal.pone.0104088)
Supplement: Table S1 — Gray matter volume differences between WS (N = 42) and TD (N = 40) groups. A custom template was used. p = 0.05 family-wise error (FWE), extent threshold (ET) = 100. (DOCX) [file pone.0104088.s005.docx]

| **Table S1.** Gray matter volume differences between WS (N=42) and TD (N=40) groups. | | | | | | | | |
| --- | --- | --- | --- | --- | --- | --- | --- | --- |
|  | **Region** | **Brodmann Area** | **Talairach Coordinates** | | | **T value** | **P value (FWE)** | **Cluster Size** |
|  |  |  | **X** | **Y** | **Z** |  |  |  |
| **WS > TD** | | | | | | | | |
|  | Rt Claustrum, Insula, Superior Temporal Gyrus | 13 | 34 | -22 | 3 | 9.35 | <0.001 | 1071 |
|  |  |  | 32 | -15 | 15 | 8.45 | <0.001 |  |
|  |  |  | 30 | -22 | 11 | 7.34 | <0.001 |  |
|  | Rt Middle, Superior Frontal Gyri | 11 | 20 | 38 | -13 | 8.97 | <0.001 | 4355 |
|  |  |  | 28 | 33 | -10 | 5.96 | 0.001 |  |
|  |  |  | 33 | 36 | -19 | 5.82 | 0.002 |  |
|  | Rt Superior Temporal, Angular, Supramarginal Gyri | 39 | 39 | -54 | 29 | 8.62 | <0.001 | 3356 |
|  | Rt Parahippocampal Gyrus, Caudate Tail | 30 | 16 | -39 | 8 | 8.54 | <0.001 | 2436 |
|  |  |  | 34 | -38 | 3 | 6.28 | <0.001 |  |
|  | Bi Anterior Cerebellum, Brainstem (Pons) |  | 9 | -44 | -29 | 8.42 | <0.001 | 4790 |
|  |  |  | 6 | -49 | -24 | 7.66 | <0.001 |  |
|  |  |  | -2 | -50 | -22 | 6.64 | <0.001 |  |
|  | Lt Parahippocampal Gyrus, Caudate Tail, Posterior Cingulate | 30,29 | -17 | -40 | 7 | 8.26 | <0.001 | 3118 |
|  |  |  | -32 | -39 | 3 | 7.07 | <0.001 |  |
|  |  |  | -10 | -43 | 12 | 6.63 | <0.001 |  |
|  | Rt Fusiform Gyrus | 19 | 34 | -67 | -6 | 6.95 | <0.001 | 2927 |
|  | Lt Insula, Superior Temporal, Transverse Gyri, Claustrum | 13,41 | -33 | -26 | 4 | 6.78 | <0.001 | 194 |
|  | Bi Posterior Cerebellum, Pyramis, Uvula |  | 21 | -71 | -28 | 6.77 | <0.001 | 4990 |
|  |  |  | -21 | -72 | -26 | 6.35 | <0.001 |  |
|  |  |  | -15 | -69 | -30 | 5.96 | 0.001 |  |
|  | Rt Precuneus | 31,7 | 19 | -73 | 26 | 6.49 | <0.001 | 1412 |
|  | Lt Middle, Superior Temporal, Angular, Supramarginal Gyri | 39 | -35 | -60 | 28 | 6.4 | <0.001 | 2399 |
|  | Rt Superior Temporal Gyrus | 22 | 61 | -15 | 4 | 6.38 | <0.001 | 1155 |
|  |  |  | 64 | -32 | 10 | 5.21 | 0.019 |  |
|  | Rt Inferior, Middle Temporal Gyri | 37 | 52 | -46 | -3 | 6.33 | <0.001 | 759 |
|  | Lt Fusiform, Middle Occipital Gyri | 19, 37 | -36 | -69 | -8 | 6.3 | <0.001 | 2370 |
|  |  |  | -36 | -59 | -7 | 6.16 | 0.001 |  |
|  | Bi Anterior Cerebellum (Culmen), Right Fusiform Gyrus |  | 1 | -49 | 0 | 6.27 | <0.001 | 623 |
|  | Lt Orcotal, Inferior, Middle Frontal Gyri | 47,11 | -26 | 21 | -17 | 6.18 | 0.001 | 1439 |
|  |  |  | -16 | 33 | -17 | 5.62 | 0.004 |  |
|  |  |  | -24 | 39 | -10 | 5.62 | 0.004 |  |
|  | Rt Middle Temporal, Occipital Gyri | 39 | 39 | -67 | 18 | 6.14 | 0.001 | 876 |
|  | Lt Cuneus, Precuneus | 18,31 | -17 | -72 | 21 | 6.12 | 0.001 | 1222 |
|  | Rt Cerebellar Tonsil |  | 12 | -55 | -34 | 5.87 | 0.002 | 119 |
|  | Rt Postcentral Gyrus | 43 | 52 | -16 | 22 | 5.83 | 0.002 | 289 |
|  | Lt Orbital, Middle, Superior Frontal Gyri | 11 | -32 | 37 | -20 | 5.74 | 0.003 | 275 |
|  | Lt Inferior Parietal Lobule, Postcentral Gyrus | 40,2 | -57 | -22 | 28 | 5.31 | 0.013 | 101 |
| **TD > WS** | | | | | | | | |
|  | Lt Intraparietal Sulcus, Precuneus | 7 | -24 | -60 | 32 | 7.47 | <0.001 | 828 |
|  | Rt Inferior Frontal Gyrus | 47 | 31 | 27 | -2 | 6.88 | <0.001 | 811 |
|  | Rt Intraparietal Sulcus, Precuneus | 7 | 28 | -61 | 33 | 6.75 | <0.001 | 656 |
|  | Bi Thalamus |  | -1 | -4 | 4 | 6.38 | <0.001 | 661 |
|  |  |  | 9 | 0 | 8 | 5.21 | 0.019 |  |
|  | Rt Precuneus, Paracentral Lobule | 7,5 | 14 | -37 | 50 | 6.05 | 0.001 | 511 |
|  | Right Insula | 13 | 35 | 15 | 7 | 5.66 | 0.004 | 393 |
|  |  |  |  |  |  |  |  |  |
