# Supplementary material for: Mapping Genetically Controlled Neural Circuits of Social Behavior and Visuo-Motor Integration by a Preliminary Examination of Atypical Deletions with Williams Syndrome
Source: PLoS One. 2014 Aug 8;9(8):e104088. doi: 10.1371/journal.pone.0104088 (PMC4126723; doi:10.1371/journal.pone.0104088)
Supplement: File S1 — Textual supporting information. (DOC) [file pone.0104088.s006.doc]

**SUPPORTING MATERIAL**

**SUPPORTING METHODS**

## ***Statistical Analysis Comparing WS and TD Individuals***

### We first examined regional gray matter volume and density differences between WS and TD individuals in the 1.5T data (42 WS vs. 40 TD). For gray matter volume, since the scaling of affine normalization was applied to the data themselves during preprocessing (m0* images in VBM5.1), it was not necessary to include total gray matter volume as a nuisance variable in the general linear models. Gray matter volume was therefore compared using analysis of variance (ANOVA). For gray matter density, analysis of covariance (ANCOVA) was performed covarying out globals and total gray matter volume. The spatial patterns of significant clusters for regional gray matter volume and density were very similar, with regional gray matter density generally showing greater levels of significance than regional gray matter volume (Figure S1). The results generally replicated the results from which used SPM99, examined gray matter density and used linear warping only for normalization in the same individuals. We compared MR images using standard SPM5 templates and custom templates and the results were also very similar (Figure S1). Finally, within the regions that showed significant difference between WS and TD groups, we examined whether similar differences existed in the data from 30 WS and 14 TD scanned with different parameters on a 3.0T MRI scanner, and the results were very similar (Figure S2).

Statistical threshold of p = 0.05 FWE corrected with an extent threshold of 100 was employed. Brain regions with significant effects are reported based on their anatomical landmarks overlaid on the custom template and several sample WS and TD images from this study. While it is not necessarily valid to report Talairach coordinates and Brodmann Areas because of the use of custom templates, we report these as reference for comparisons with other studies. The reported Talairach coordinates were converted from MNI space using the mni2tal function (<http://www.mrc-cbu.cam.ac.uk/Imaging/Common/mnispace.shtml>).

**SUPPORTING RESULTS**

**Behavioral: Comparing WS and TD individuals**

WS individuals showed significantly reduced IQ and visuo-spatial abilities, and significantly higher social approachability scores as compared to TD controls (all p’s<0.05). Please see **Table 1** for their mean scores, statistics, and **Table 2** for scores of partial deletion cases.

**Neuroanatomical: Comparing WS and TD individuals**

First examining VBM gray matter volume results from 1.5T MRI using custom templates, we found that volumes surrounding the bilateral intraparietal sulcus (IPS) were reduced relative to total gray matter volume in WS compared to TD, while other regions, including bilateral insular, orbitofrontal, inferior parietal, temporal, fusiform, parahippocampal and cerebellar regions, were increased relative to total gray matter volume (**Figure S1,** **Table S1**). Bilateral amygdala delineated manually, also had greater relative volume in WS compared to TD controls (**Table 1, Figure 2B**). The spatial patterns of significant clusters for regional gray matter volume and density were very similar, with regional gray matter density generally showing greater levels of significance than regional gray matter volume (**Figure S1**). The results replicated the findings from which used SPM99, examined gray matter density and used linear warping only for normalization in the same individuals. We compared MR images using standard SPM5 templates and custom templates and the results were also very similar (**Figure S1**). Finally, within the regions that showed significant difference between WS and TD groups, we examined whether similar differences existed in the 3.0T MRI data from 30 WS and 14 TD scanned with different parameters, and the results were very similar (**Figure S2**).

**Neuroanatomical: AWSdel individuals**

AWSdel results comparing standard gray matter volume (**Figure 2A**) and density (**Figure S3**), and 1.5T (**Figure 2A**) and 3.0T (**Figure S4**) showed no differences in results.
